# Prognostic factors for first recurrence following meningioma surgery

**DOI:** 10.3892/mi.2024.213

**Published:** 2024-12-31

**Authors:** George Fotakopoulos, Vasiliki Epameinondas Georgakopoulou, Demetrios A. Spandidos, Efthalia Angelopoulou, Apostolos-Alkiviadis Menis, Nikolaos Trakas, George Alexiou, Spyridon Voulgaris

**Affiliations:** 1 Department of Neurosurgery, General University Hospital of Larissa, 41221 Larissa, Greece; 2 Department of Pathophysiology, Laiko General Hospital, National and Kapodistrian University of Athens, 11527 Athens, Greece; 3 Laboratory of Clinical Virology, School of Medicine, University of Crete, 71003 Heraklion, Greece; 4 1st Department of Neurology, Eginition Hospital, National and Kapodistrian University of Athens, 11528 Athens, Greece; 5 Intensive Care Unit, General University Hospital of Larissa, 41221 Larissa, Greece; 6 Department of Biochemistry, Sismanogleio Hospital, 15126 Athens, Greece; 7 Department of Neurosurgery, School of Medicine, University of Ioannina, 45500 Ioannina, Greece

**Keywords:** meningioma, recurrence, Simpson grade, MIB-1

## Abstract

The present study investigated the role of the Simpson grade system, MIB-1 immunohistochemical marker, meningioma location and grade in the risk of recurrence. Between January, 2008 and January, 2018, the present study retrospectively evaluated all patients undergoing craniotomy for the resection of a histopathologically confirmed meningioma. Patients with neurofibromatosis, acoustic neurinomas and radiation treatment prior to surgery were excluded. After applying the exclusion criteria, 103 patients were included in the study. Following a mean follow-up period of 67.3±33 months, there were 12 cases (11.6%) of tumor recurrence. No significant association between meningioma recurrence and age, sex, or tumor location was found. When comparing the risk of recurrence between Simpson grades I, II, III and IV excisions, and between Simpson grade V, the difference was statistically significant. When comparing WHO grade I and II meningioma vs. grade III, the difference was significant. MIB-1 LI >3% exhibited a trend towards a significant association with the risk of recurrence. On the whole, the present study demonstrates that the Simpson grade is associated with the risk of recurrence. Patients with tumors with an MIB-1 index >3% may also be at a risk of recurrence. Notably, the present study proposed that in the case of recurrence, this is more likely to occur in an interval of 5.5 years following surgical intervention.

## Introduction

Since 1957, when Simpson classified the extent of resection of meningiomas into five subdivisions, various efforts have been made to achieve a more extensive surgical excision. The post-operative recurrence rates of patients with meningiomas were associated with the extent of resection, and when the patients survived for 6 months following surgery with Simpson grades I, II, III and IV, the recurrence rates were 9, 16, 29 and 39%, respectively (1-4). However, the latest monitoring devices, surgical equipment such as the Isocool^®^ bipolar forceps or the cavitronic ultrasonic surgical aspirators (CUSA) device, and diagnostic procedures such as magnetic resonance imaging (MRI) are currently commonly used, markedly improving the extent of resection of meningiomas, and thus reducing the recurrence rate. However, the necessity of post-operative radiation therapy for World Health Organization (WHO) grade I meningiomas based on the Simpson grade system still remains uncertain. According to certain reports, the use of the proliferation tumor marker MIB-1 may be useful (5-10). However, the association between the location of the meningioma and recurrence warrants further and more in-depth investigations.

The present study investigated the role of the Simpson grade system, meningioma location and grade in the risk of recurrence, and aimed to assess the proliferative index in a series of surgically removed meningiomas using immunohistochemical methods with immunohistochemical marker (MIB-1) labeling indices (LI) associating this index with clinical, radiological and histological factors.

## Patients and methods

### Study protocol and patients

Between January, 2008 and January, 2018, the present study retrospectively evaluated all patients undergoing craniotomy for resection of a histopathologically confirmed meningioma from the General University Hospital of Ioannina (Ioannina, Greece). A total of 103 patients were derived into two groups as follows: Group A (91 patients) without recurrence and group B (12 patients) that had detected a meningioma recurrence. When the patient underwent multiple surgeries, only the data from the first surgery were included. Patients with neurofibromatosis, acoustic neurinomas and radiation treatment prior to surgery were excluded. In addition, patients with any other intracranial tumor history or recurrent meningioma whose primary surgery was performed at another institute were not included. The present study received institutional ethical approval from the General University Hospital of Ioannina (reference no. 9769/24-6-2019). The present study was performed in line with the Declaration of Helsinki (1995; as revised in Edinburgh 2000). Written informed consent was obtained from all the included patients.

### Study outcomes

The study end-points comprised neurological improvement as the main outcome on the quality of life of patients. The follow-up period for the patients was 6 to 123 months.

### Immunohistochemistry

Immunohistochemistry was performed according to standardized methods on paraffin-embedded sections of meningioma specimens. The thickness of the sections used was 4 µm. Deparaffinized tissue sections were treated with 10% hydrogen peroxide (H_2_O_2_) in methanol at room temperature for 4 min. Antigen retrieval was performed by autoclave for 10 min at 120˚C. The sections were incubated in 8% skim milk-Tris-buffered saline at 37˚C for 40 min to prevent non-specific reactions and subsequently at 4˚C overnight with the following primary antibodies: Mouse anti-cytokeratin (AE1/AE3; cat. no. ab961; Abcam.), mouse anti-vimentin (cat. no. sc-6260; Santa Cruz biotechnologies, Inc.), rabbit anti-claudin-1 (polyclonal; cat. no. SAB4503546; MerckMillipore), mouse anti-E-cadherin (cat. no. ab287970; Abcam), mouse anti-β-catenin (cat. no. 14-2567-82; Thermo Fisher Scientific, Inc.), mouse anti-N-cadherin (cat. no. ab98952; Abcam), and mouse anti-Ki-67 (cat. no. MBS9700363; MyBioSource). The secondary antibodies used were anti-mouse or anti-rabbit Envision horseradish peroxidase-labelled polymer (9003-99-0; Merck Millipore) which were then applied at 37˚C for 40 min. Finally, the reactions were visualized with 0.05% 3-3'-diaminobenzidine and 0.03% hydrogen peroxide in Tris-hydrochloric acid buffer, followed by a counterstain with Mayer's hematoxylin at a temperature of 4˚C for ~3 sec. The sections were viewed under an Olympus BX60 fluorescent microscope with appropriate filters (Olympus Corporation), and those exhibiting 90% nuclei with signals were evaluated, with 100 to 200 intact nonoverlapping nuclei scored for the number of fluorescent signals.

### Statistical analysis

Statistical analyses were performed using the Statistical Package for the Social Sciences (SPSS 11; SPSS, Inc.). Fisher's exact test was used to compare the groups, and continuous data were compared using the Mann-Whitney U test. Receiver operating characteristic (ROC) analysis was used to reveal the factors that are related to first recurrence and affect the outcomes of patients following meningioma surgery. A P-value <0.05 was considered to indicate a statistically significant difference.

## Results

After applying the exclusion criteria, 103 patients (30 males and 73 females) were included in the study. The mean and the median ages of the patients were 62.6 and the 65.5 years, respectively, (range, 22-83 years). A summary of the patient data is presented in Table I. Following a mean follow-up period of 67.3±33 months (range, 6 to 123 months), there were 12 cases (11.6%) of tumor recurrence. There was no significant difference between meningioma recurrence risk and age, sex, or tumor location. When comparing the risk of recurrence between Simpson grade V, WHO grade III, histology and the recurrence interval, the difference was statistically significant (P<0.05; Table I).

Subsequently, univariate analysis for neurological improvement revealed that there was a statistically significant difference in the following patient parameters: WHO grade III, histology (anaplastic or atypical), MIB-1 LI >3, Simpson grade V and the recurrence interval between the participants who were operated on for meningiomas (P<0.05, Table II). Multivariate analysis (Table III) revealed that among the aforementioned parameters, the recurrence interval and Simpson grade V were independent factors associated with neurological improvement during follow-up with P<0.05 and P=0.049, respectively, and the combination of the WHO grade III, histology (anaplastic or atypical), MIB-1 LI >3, and Simpson grade I parameters can predict the meningioma recurrence.

**Figure 1:**
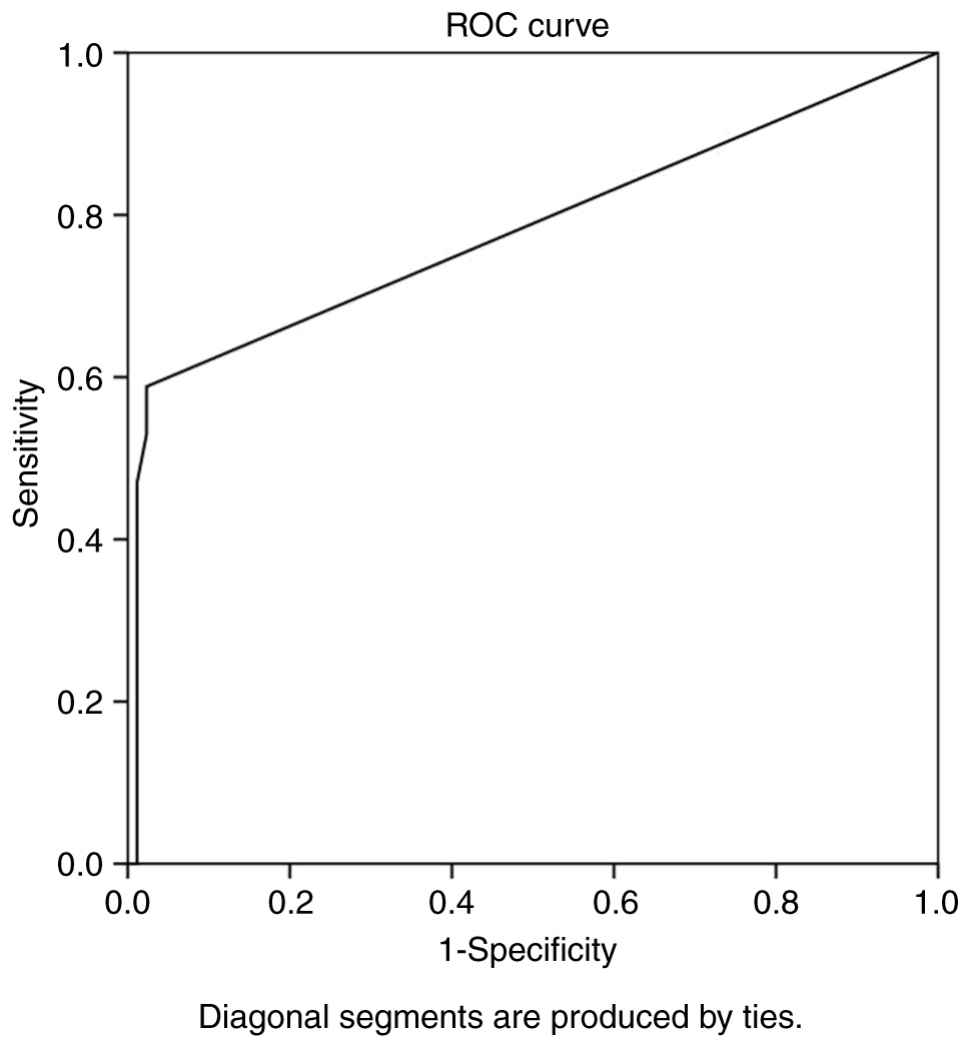
ROC curve for recurrence Interval, predicting meningioma recurrence event during follow-up. Area under the ROC curve, 0.781. ROC, receiver operating characteristic.

**Figure 2:**
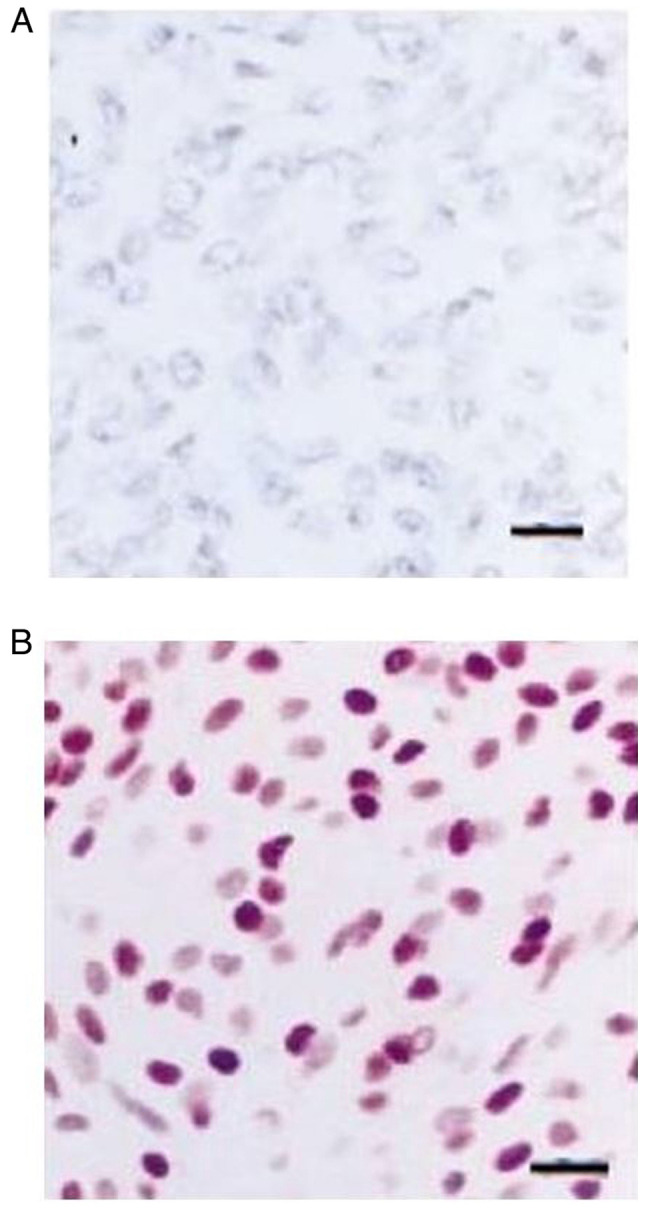
(A) Immunohistochemical analysis of a 67-year-old male exhibiting a lack of expression following staining for epithelial membrane antigen in an anaplastic meningioma; scale bar, 20 µm. (B) Immunohistochemical analysis of a 64-year-old female exhibiting partially positive staining for epithelial membrane antigen; scale bar, 20 µm.

Overall, ROC analysis demonstrated that the recurrence interval exhibited the optimal performance to predict meningioma reappearance, as evaluated by an area under the curve standard error [AUC(SE)] of [0.781 (0.076) and (P=0.001)] and [0.633 (0.930)] (Table III and Fig. 1). In addition, ROC analysis demonstrated that, among the variables, an interval from surgical removal at 5.5 years with 89% sensitivity and 98% specificity exhibited a better dispersion to predict tumor recurrence, as evaluated by an area under the curve standard error [AUC(SE)] of [0.781 (0.076)] and (P=0.001)] Table III and Fig. 1. Immunohistochemical analysis revealed partially positive staining for epithelial membrane antigen for non-anaplastic meningiomas, but a lack of expression following staining for epithelial membrane antigen in an anaplastic meningioma (Fig. 2).

## Discussion

The results of the present study suggested that simple decompression with or without biopsy and surgical resection of meningiomas (Simpson grade V) was one of the main factors in predicting the risk of meningioma recurrence. Overall, WHO grade III, histology (anaplastic or atypical), and MIB-1 LI >3 parameters were not independent factors in predicting the recurrence of meningiomas (Fig. 2). This cohort proposes that the role of a high MIB-1 index (>3%) as a key factor associated with meningioma recurrence is limited compared with the literature (20-24) and only the combination with WHO grade III histology (anaplastic or atypical) may increase the risk of tumor recurrence. In addition, instead of the most effective and widely accepted treatment among neurosurgeries worldwide, namely the Simpson grade I resection for reducing tumor recurrence (11-14), the present study suggests that particularly for surgical resection, according to Simpson grade I, II, III and IV, the recurrence rate is the same, but markedly changes when the Simpson grade is V (only decompression with or without biopsy). This means that even a subtotal tumor resection (Simpson grade IV), independent of the tumor location, could not affect the recurrence rate. If the histological type is not WHO III or anaplastic/atypical, simple tumor removal is sufficient in the majority of cases. Of note, the present study proposed that if the recurrence occurs, it is more likely to occur in an interval after 5.5 years of surgical intervention.

For a number of years, the gross total surgical resection (GTR) of meningiomas with the affected dura and underlying bone (Simpson grade I) was the most effective and widely accepted treatment among neurosurgeries worldwide, reducing tumor recurrence (11-14). On the other hand, there are new reports demonstrating that simply removing the entire tumor, even if small areas are left close to critical structures, achieves the same result compared with the more aggressive resection of the dura and underlying bone (2). This is the most effective treatment mainly for the optic nerve sheath meningiomas, where fractionated radiotherapy complements the surgery (15). In the present study on 103 cases undergoing surgery, 12 cases (11.6%) of recurrent meningiomas were elicited; 2 of these cases (1.9%) were located at the convexity, despite the *en block* tumor removal. This is the reason why the surgical plan for meningiomas must be revised based on the collective results of the meningiomas location, Simpson grade scale, and MIB-1 index in order to have a better understanding of the different factors that may play a role in tumor recurrence. For example, as regards the location of meningiomas, Zhang *et al* (16) reported that petroclival meningiomas, due to deep location and for being adjacent to neurovascular structures are generally considered to be associated with a high rate of recurrence.

Post-operative radiation therapy, pre-operative endovascular embolization, intraoperative monitoring, the widespread use of new surgical equipment such as the CUSA, microscope, ISOCOOL bipolar device and the benefits of the MRI on the definition of recurrence have changed the importance of the Simpson grade system in the modern era. The goal of meningiomas surgery is currently to eliminate, to the greatest extent possible, tumors without efforts to achieve a higher Simpson grade score by removing the dura or the bone. This is more important in skull-base meningiomas (2). On the other hand, particularly for convexity meningiomas, the Simpson grade I resection appears to be the main target during surgery, reducing the tumor recurrence rate (3). In addition to the same meningioma subtype, other research has reported that despite the entire tumor removal, the pial participation and/or vascular attachments play a crucial role in recurrence (4). When analyzing the association between Simpson grade categories of meningiomas and recurrence, it was established that there were statistically significant differences among Simpson grade I, II, III and IV vs. grade V in the groups, with no statistically significant findings between the Simpson grade I, II, III and IV groups. This indicates that, particularly for surgical resection, according to Simpson grade I, II, III and IV, the recurrence rate is the same but markedly changes when the Simpson grade becomes V.

It is important to note that currently, the ability to correctly discriminate between higher-grade meningiomas and thus to rule them out of any studies for benign meningiomas, compared to the past 20-30 years, is more valuable (17-21). The present study evaluated 103 cases undergoing surgery for intracranial meningiomas of WHO I, II and grade III. After 6-123 months of follow-up, 12 cases (11.6%) with tumor recurrence were found. When analyzing the association between the WHO grade categories of meningiomas and recurrence, it was established that there were statistically significant differences among WHO I vs. II and III and WHO I via III groups, with no statistically significant findings between the WHO I and II groups. This means that, particularly for the histological types WHO I and II, the recurrence rate is the same, but markedly changes when the grade of proliferation becomes III.

It has been well recognized through several studies that a high MIB-1 index (>3%) is associated with the recurrence of meningioma (21-25). In this cohort, the role of MIB-1 as an important factor associated with meningioma recurrence is limited. The present study found a trend towards a significant association between MIB-1 and the recurrence of meningioma. In the present study, the MIB-1 LI >3 parameter was not an independent factor to predict the recurrence of meningioma; only the combination with WHO grade III histology (anaplastic or atypical) may increase the risk of tumor recurrence.

The present study has certain limitations which should be mentioned. One limitation was that in clinical practice, the rate of recurrence varies according to the location of the meningioma; meningiomas located at the skull base, particularly those in the foramen magnum, petroclival region, anterior skull base and sphenoid ridge, are more prone to recurrence, as it is difficult to achieve Simpson grade I resection for meningiomas located in these areas; thus, due to the small number of cases, the present study was unable to reflect this point. In addition, the results demonstrated that the Simpson grade was associated with the risk of recurrence; however, due to the small number of excisions of Simpson grades ≥3, there may have been a significant deviation in the results of recurrence. In addition, the follow-up period of 6-123 months, may have been too short for the recurrence of meningioma, as meningiomas often occur following a long period of time (>10 years) post-surgery. Finally, the small number of 12 cases of recurrence, may have caused bias in the results and thus to the excluded factors, such as MIB-1 LI, Simpson grade, WHO grade, which are recognized to affect the post-operative recurrence of meningiomas.

In conclusion, while for the histological types WHO I and II, the recurrence rate does not differ, when the grade of proliferation becomes III, the behavior of the meningioma markedly changes. The GTR with all affected dura and the underlying bone (Simpson grade I) had a significant sorter risk for recurrence, but with the same recurrence rate compared with Simpson grade II, III or IV resection. This may demonstrate that if the histological type is not WHO III or anaplastic/atypical, the simple removal of the tumor is sufficient in most cases. However, the risk of recurrence markedly changes when the Simpson grade of proliferation becomes V. Notably, the present study suggests that if recurrence occurs, it is more likely to occur at an interval of 5.5 years following surgical intervention.

The role of MIB-1 as a key factor associated with meningioma recurrence is limited, which indicates that the risk of tumor recurrence is low.

The present study suggests that the surgical plan for meningiomas needs to be revised on the basis of the combined influence of the meningiomas histological type, Simpson grade scale, MIB-1 LI value, and eventually, its location in order to achieve improved outcomes with aggressive surgical and post-surgical treatment, often recommending radiation therapy. In addition, further multi-center collaborative studies are required in order to obtain a more in-depth understanding of the different factors that may play a role in the recurrence of meningiomas.

## Figures and Tables

**Table I:** Baseline demographic characteristics of the patients.

Parameters	All patients, n=103 (100%)	Group A, n=91 (88.3%)	Group B, n=12 (11.6%)	P-value
Age, mean ± SD (years)	62.6±12.6	62.3±12.3	65.0±14.7	0.318
Sex, n (%)				0.738
Male	30 (29.1)	27 (26.2)	3 (2.9)	
Female	73 (70.9)	64 (62.1)	9 (8.7)	
Anticoagulant, n (%)				0.442
Yes	45 (43.6)	41 (39.8)	4 (3.8)	
No	58 (56.3)	50 (48.5)	8 (7.7)	
Diabetes, n (%)				0.281
Yes	31(30)	29 (28.1)	2 (1.9)	
No	72 (69.9)	62 (60.1)	10 (9.7)	
Hypertension, n (%)				0.346
Yes	29 (28.1)	27 (26.2)	2 (1.9)	
No	74 (71.8)	64 (62.1)	10 (9.7)	
History of seizures, n (%)				0.513
Yes	25 (24.2)	23 (22.3)	2 (1.9)	
No	78 (75.7)	68 (66.0)	10 (9.7)	
WHO grade				
I, n (%)	80 (76.6)	75 (72.8)	5 (4.8)	0.002^a^
II, n (%)	13 (12.6)	11 (10.6)	2 (1.9)	0.653^a^
III, n (%)	10 (9.7)	5 (4.8)	5 (4.8)	0.001^a^
Location				0.223
Convexity, n (%)	70 (67.9)	64 (62.1)	6 (5.8)	
Cerebellum, n (%)	4 (3.8)	3 (2.9)	1 (0.9)	
Parasagittal, n (%)	21 (20.3)	16 (15.5)	5 (4.8)	
Sella turcica, n (%)	6 (5.8)	6 (5.8)	0 (0)	
Multiple, n (%)	2 (1.9)	2 (1.9)	0 (0)	
Histology				0.001
Anaplastic or atypical, n (%)	12 (11.6)	4 (3.8)	8 (7.7)	
Other, n (%)	91 (88.3)	77 (74.7)	14 (13.5)	
MIB-1 LI, n (%)				0001
>3	28 (27.1)	18 (17.4)	10 (9.7)	
<3	75 (72.8)	73 (70.8)	2 (1.9)	
Simpson grade				
I, n (%)	69 (66.9)	68(66)	1 (0.9)	0.001^a^
II, n (%)	21 (20.3)	16 (15.5)	5 (4.8)	0.052^a^
III, n (%)	5 (4.8)	3 (2.9)	2 (1.9)	0.043^a^
IV, n (%)	1 (0.9)	1 (0.9)	0 (0)	0.715^a^
V, n (%)	8 (7.7)	4 (3.8)	4 (3.8)	0.001^a^
Recurrence interval, mean ± SD (years)	0.8±2.7	0	7.3±4.1	0.001

^a^The P-value for each grade corresponds to the comparison between this grade and all remaining grades. MIB-1 LI, immunohistochemical marker labeling indice; WHO, World health organization); SD, standard deviation; MIB-1, cell proliferation marker labeling indice (LI).

**Table II:** Univariate analysis for neurological improvement.

Parameters	No improvement, n=17 (16.5%)	With improvement, n=86 (83.4%)	P-value
Age, mean ± SD (years)	66.7±13	61.8±12	0.093
Sex, n (%)			0.231
Male	7 (6.7)	23 (22.3)	
Female	10 (9.7)	63 (61.1)	
Anticoagulant, n (%)			0.400
Yes	9 (8.7)	36 (34.9)	
No	8 (7.7)	50 (48.5)	
Diabetes, n (%)			0.946
Yes	5 (4.8)	26 (25.2)	
No	12 (11.6)	60 (58.2)	
Hypertension, n (%)			0.100
Yes	2 (1.9)	27 (26.2)	
No	15 (14.5)	59 (57.2)	
History of seizures, n (%)			0.938
Yes	4 (3.8)	21 (20.3)	
No	13 (12.6)	65 (63.1)	
WHO grade			
I, n (%)	10 (9.7)	69 (66.9)	0.056^a^
II, n (%)	3 (2.9)	10 (9.7)	0.495^a^
III, n (%)	4 (3.8)	6 (5.8)	0.035^a^
Location			0.621
Convexity, n (%)	11 (10.6)	59 (57.2)	
Cerebellum, n (%)	1 (0.9)	3 (2.9)	
Parasagittal, n (%)	5 (4.8)	16 (15.5)	
Sella turcica, n (%)	0 (0)	6 (5.8)	
Multiple, n (%)	0 (0)	2 (1.9)	
Histology			0.005
Anaplastic or atypical, n (%)	8 (7.7)	14 (13.5)	
Other, n (%)	9 (8.7)	72 (69.9)	
MIB-1 LI, n (%)			0.006
>3	10 (9.7)	18 (17.4)	
<3	7 (6.7)	68 (66.0)	
Simpson grade			
I, n (%)	8 (7.7)	61 (59.2)	0.056^a^
II, n (%)	4 (3.8)	17 (16.5)	0.725^a^
III, n (%)	1 (0.9)	4 (3.8)	0.829^a^
IV, n (%)	0 (0)	1 (0.9)	0.655^a^
V, n (%)	4 (3.8)	4 (3.8)	0.008^a^
Recurrence interval, mean ± SD (years)	3.9±4.0	0.8±2.7	0.001

^a^The P-value for each grade corresponds to the comparison between this grade and all remaining grades. MIB-1 LI, immunohistochemical marker labeling indice; WHO, World health organization); SD, standard deviation; MIB-1, cell proliferation marker labeling indice (LI).

**Table III:** Multivariate analysis and ROC analysis for neurological improvement.

A, Multivariate analysis				
			95% CI for Exp (B)	
Parameter	P-value	Exp (B)	Lower	Upper
WHO grade, III	0.351	0.108	-0.151	0.421
Histology, anaplastic or atypical	0.249	-0.132	-0.325	0.085
MIB-1 LI >3	0.396	-0.082	-0.229	0.091
Simpson grade, V	0.049	-0.173	-0.478	0.001
Recurrence interval	0.001	-0.437	-0.088	-0.031
B, ROC analysis				
Parameter	P-value	Area	Std. Error	CI (95%) lower-upper
Recurrence interval	0.001	0.781	0.076	0.633-0.930

SD, standard deviation; CI, conﬁdence interval; MIB-1 LI, immunohistochemical marker labeling indice; WHO, World health organization; ROC, receiver operating characteristic.

## Data Availability

The data generated in the present study may be requested from the corresponding author.
